# Crystal structure and DFT study of (*E*)-*N*-[2-(1*H*-indol-3-yl)eth­yl]-1-(anthracen-9-yl)methanimine

**DOI:** 10.1107/S2056989017011483

**Published:** 2017-08-11

**Authors:** Md. Serajul Haque Faizi, Necmi Dege, Sergey Malinkin, Tetyana Yu. Sliva

**Affiliations:** a Department of Chemistry, College of Science, Sultan Qaboos University, PO Box 36 Al-Khod 123, Muscat, Sultanate of Oman; b Ondokuz Mayıs University, Arts and Sciences Faculty, Department of Physics, 55139 Atakum-Samsun, Turkey; c Department of Chemistry, National Taras Shevchenko University of Kiev, 64/13 Volodymyrska Street, City of Kyiv 01601, Ukraine

**Keywords:** crystal structure, anthracene, indole, Schiff base, tryptamine, methanamine, N—H⋯π inter­actions, C—H⋯π inter­actions

## Abstract

The title compound, C_25_H_20_N_2_, (**I**), was synthesized from the condensation reaction of anthracene-9-carbaldehyde and tryptamine in dry ethanol. The indole ring system (r.m.s. deviation = 0.016 Å) makes a dihedral angle of 63.56 (8)° with the anthracene ring (r.m.s. deviation = 0.023 Å). There is a short intra­molecular C—H⋯N inter­action present, and a C—H⋯π inter­action involving the two ring systems. In the crystal, the indole H atom forms an inter­molecular N—H⋯π inter­action, linking mol­ecules to form chains along the *b*-axis direction. There are also C—H⋯π inter­actions present, involving the central and terminal rings of the anthracene unit, linking the chains to form an overall two-dimensional layered structure, with the layers parallel to the *bc* plane. The density functional theory (DFT) optimized structure, at the B3LYP/6-311 G(d,p) level, is compared with the experimentally determined mol­ecular structure in the solid state.

## Abstract 2

In the title compound, the indole ring system makes a dihedral angle of 63.56 (8)° with the plane of the anthracene ring. The conformation about the C=N bond of the –CH_2_–CH_2_–N=CH– bridge linking the two units is *E*. In the crystal, the indole H atom is involved in an inter­molecular N—H⋯π inter­action with the benzene ring of the indole group, leading to the formation of chains along [010].

## Chemical context   

Tryptamine is a biogenic serotonin-related indo­amine and is the deca­rboxylation product of the amino acid tryptophan. 2-(1*H*-Indol-3-yl)ethanamine is an alkaloid found in plants and fungi and is a possible inter­mediate in the biosynthetic pathway to the plant hormone indole-3-acetic acid (Takahashi, 1986 ▸). It is also found in trace amounts in the mammalian brain, possibly acting as a neuromodulator or neurotransmitter (Jones, 1982 ▸). There are seven known families of serotonin receptors, which are tryptamine derivatives. All of them are neurotransmitters. Hallucinogens all have a high affinity for certain serotonin receptor subtypes and the relative hallucinogenic potencies of various drugs can be gauged by their affinities for these receptors (Glennon *et al.*, 1984 ▸; Nichols & Sanders-Bush, 2001 ▸; Johnson *et al.*, 1987 ▸; Krebs-Thomson *et al.*, 1998 ▸). The structures of many hallucinogens are similar to serotonin and have a tryptamine core. Indole analogues, especially of tryptamine derivatives, have been found to be polyamine site antagonists at the *N*-methyl-d-aspartate receptor (NMDAR; Worthen *et al.*, 2001 ▸). Indole and its derivatives are secondary metabolites that are present in most plants (such as unripe bananas, broccoli and clove), almost all flower oils (*e.g.* jasmine and orange blossoms) and coal tar (Waseem & Mark 2005 ▸; Lee *et al.*, 2003 ▸). In the pharmaceutical field, it has been discovered that it has anti­microbial and anti-inflammatory properties (Mohammad & Moutaery, 2005 ▸). The present work is part of an ongoing structural study of Schiff bases and their utilization in the synthesis of new organic and polynuclear coordination compounds, and their application in fluorescence sensors (Faizi & Sen, 2014 ▸; Faizi *et al.*, 2016 ▸). We report herein the crystal structure of (*E*)-*N*-[2-(1*H*-indol-3-yl)eth­yl]-1-(an­thra­cen-9-yl)methanimine, (**I**), and its DFT computational calculation. Calculations by density functional theory (DFT) on (**I**), carried out at the B3LYP/6-311 G(d,p) level, are compared with the experimentally determined mol­ecular structure in the solid state.
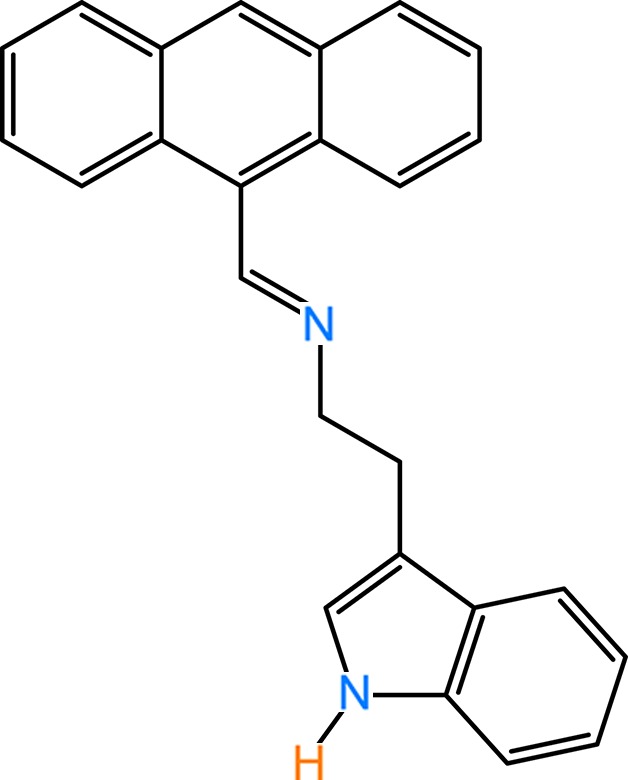


## Structural commentary   

**Figure 1:**
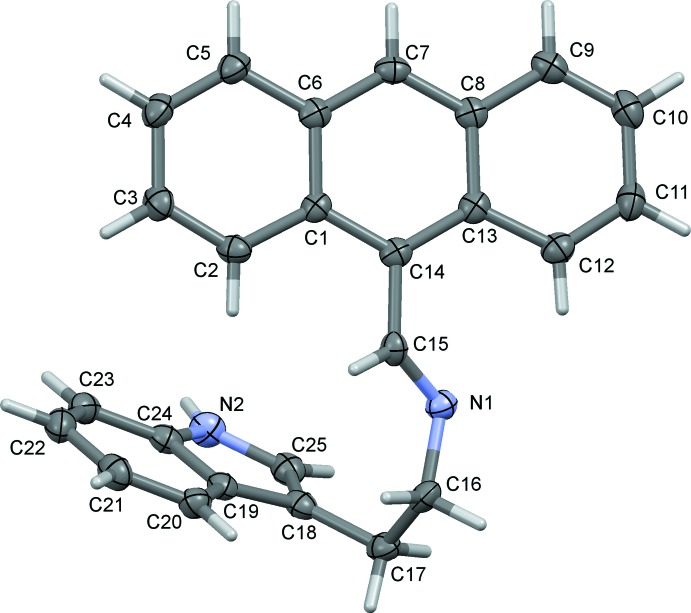
The mol­ecular structure of compound (**I**), with the atom labelling. Displacement ellipsoids are drawn at the 50% probability level.

The mol­ecular structure of compound (**I**) is illustrated in Fig. 1 ▸. The mol­ecule adopts a nonplanar geometry, with the dihedral angle between the planes of the indole and anthracene rings being 63.56 (8)°. The conformation about the azomethine C15=N1 bond [1.272 (10) Å] is *E*, with the C14—N2—C12—C13 torsion angle being 179.0 (1)°. The mol­ecule is stabilized by a weak intra­molecular hydrogen bond (C12—H12⋯N1) and a C—H⋯π inter­action (C2—H2⋯*Cg*5; *Cg*5 is the centroid of the C19–C24 ring); see Table 1 ▸.

## Supra­molecular features   

**Figure 2:**
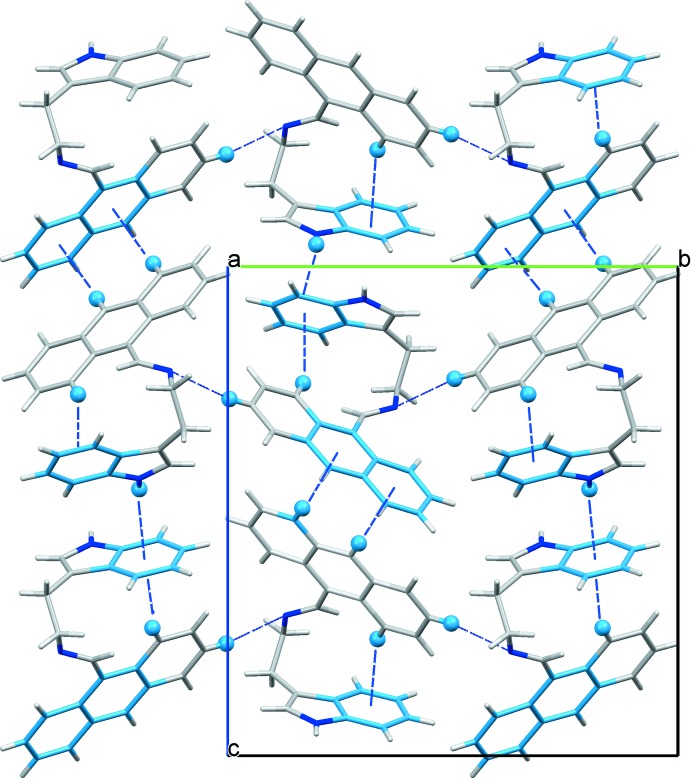
A view along the *a* axis of the crystal packing of compound (**I**), showing the layer-like structure. Weak N—H⋯π and C—H⋯π inter­actions are shown as blue dashed lines (see Table 1 ▸).

In the crystal, the indole H atom forms an inter­molecular N—H⋯π inter­action, linking mol­ecules to form chains along the *b*-axis direction (Fig. 2 ▸ and Table 1 ▸). There are also C—H⋯π inter­actions present, involving the central ring and terminal rings of the anthracene unit, linking the chains to form layers parallel to the *bc* plane (Fig. 2 ▸ and Table 1 ▸).

## Database survey   

A search of the Cambridge Structural Database (CSD, Version 5.38, update February 2017; Groom *et al.*, 2016 ▸) revealed the structures of several similar compounds containing a phenol group [(**II**) (CSD refcode FAJVIV; Rodriguez *et al.*, 1987 ▸) and (**III**) (TANNOL; Ishida *et al.*, 1992 ▸)] and nitro­benzene moieties [(**IV**), GEYPEF; Törnroos, 1988 ▸]. All compounds are 2-indole-substituted derivatives which have two aromatic units linked *via* an aliphatic chain. In (**I**), the dihedral angle between indole and anthracene rings is 63.56 (8)°, which is similar for (**III**) and (**IV**), *viz.* 71.52 and 64.21°, respectively. In compounds (**I**) and (**II**), the conformation about the azomethine C15=N1 bond is *E*.

## DFT study   

Calculations by density functional theory DFT-B3LYP, with basis set 6-311 G(d,p), of bond lengths and angles were performed. These values are compared with the experimental values for the title system (see Table 2 ▸). From these results we can conclude that basis set 6-311 G(d,p) is better suited in its approach to the experimental data.

**Figure 3:**
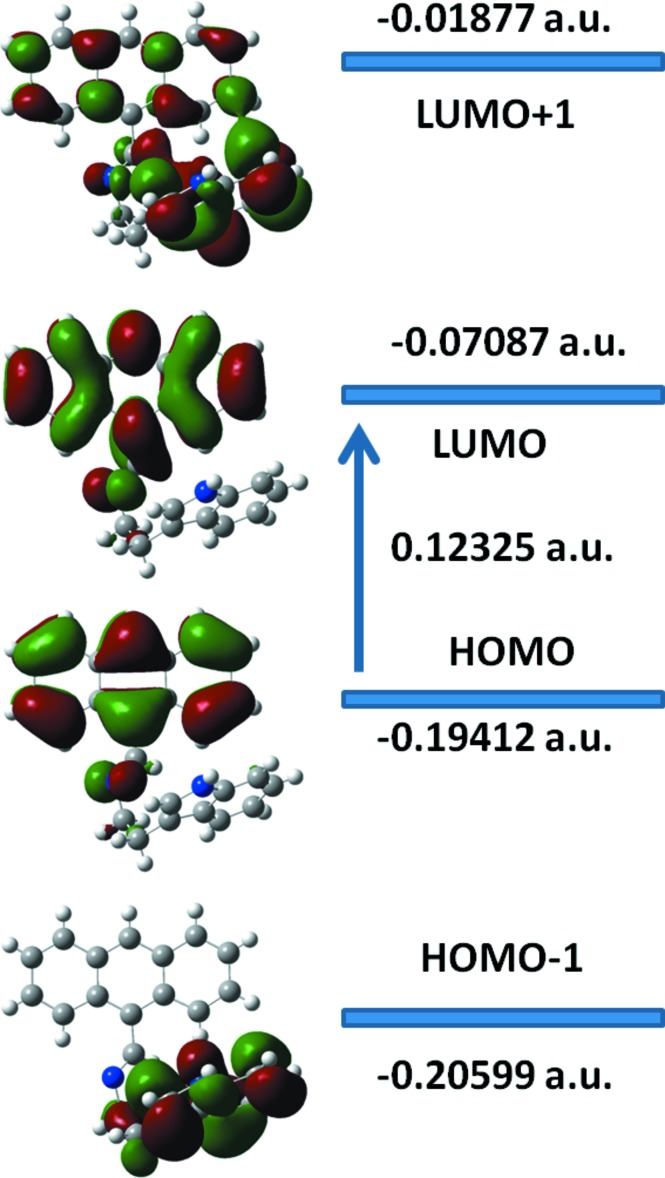
Electron distribution of the HOMO-1, HOMO, LUMO and LUMO+1 energy levels for compound (**I**).

The LUMO and HOMO orbital energy parameters are considerably answerable for the charge transfer, chemical reactivity and kinetic/thermodynamic stability of (**I**). The DFT study of (**I**) revealed that the HOMO and LUMO are localized in the plane extending from the whole anthracene ring to the indole ring, and electron distribution of the HOMO-1, HOMO, LUMO and LUMO+1 energy levels are shown in Fig. 3 ▸. The mol­ecular orbital of HOMO contains both σ and π character, whereas HOMO-1 is dominated by π-orbital density. The LUMO is mainly composed of σ density, while LUMO+1 has both σ and π electronic density. The HOMO–LUMO gap for (**I**) was found to be 0.12325 a.u. and the frontier mol­ecular orbital energies, *E*_HOMO_ and *E*_LUMO_, were found to be −0.196412 and −0.07087 a.u., respectively.

## Synthesis and crystallization   

80 mg (0.435 mmol) of 2-(1*H*-indol-3-yl)ethanamine (tryptamine) were dissolved in 10 ml of absolute ethanol. To this solution, 89 mg (0.434 mmol) of anthracene-9-carbaldehyde in 5 ml of absolute ethanol were added dropwise under stirring. The mixture was stirred for 10 min, two drops of glacial acetic acid were added and the mixture was refluxed for a further 2 h. The resulting yellow precipitate was recovered by filtration, washed several times with small portions of ice-cold ethanol and then with diethyl ether to give 140 mg (87%) of compound (**I**). Dark-yellow block-like crystals suitable for X-ray analysis were obtained within 3 d by slow evaporation of a solution in methanol.

## Refinement   

Crystal data, data collection and structure refinement details are summarized in Table 3 ▸. The N—H H atom was located from a difference-Fourier map and constrained to ride on the parent atom: N—H = 0.86 Å and *U*_iso_(H) = 1.2*U*_eq_(N). All C-bound H atoms were positioned geometrically and refined using a riding model, with C—H = 0.93–0.97 Å and *U*_iso_(H) = 1.2*U*_eq_(C).

The DFT quantum-chemical calculations were performed at the B3LYP/6-311 G(d,p) level (Becke, 1993 ▸; Lee *et al.*, 2003 ▸) as implemented in *GAUSSIAN09* (Frisch *et al.*, 2009 ▸). DFT structure optimization of (**I**) was performed starting from the X-ray geometry.

## Supplementary Material

Crystal structure: contains datablock(s) I, Global. DOI: 10.1107/S2056989017011483/su5387sup1.cif

Structure factors: contains datablock(s) I. DOI: 10.1107/S2056989017011483/su5387Isup2.hkl

CCDC reference: 1537222

Additional supporting information:  crystallographic information; 3D view; checkCIF report

## Figures and Tables

**Table 1:** Hydrogen-bond geometry (Å, °) *Cg*3, *Cg*4 and *Cg*5 are the centroids rings C1/C6–C8/C13/C14, C8–C13 and C19–C24, respectively.

*D*—H⋯*A*	*D*—H	H⋯*A*	*D*⋯*A*	*D*—H⋯*A*
C12—H12⋯N1	0.93	2.36	2.9845 (2)	124
C2—H2⋯*Cg*5	0.93	2.77	3.5505 (2)	142
N2—H2*A*⋯*Cg*5^i^	0.86	2.59	3.1855 (2)	127
C7—H7⋯*Cg*4^ii^	0.93	2.75	3.5777 (2)	148
C9—H9⋯*Cg*3^ii^	0.93	2.73	3.5077 (2)	142
C16—H16*A*⋯*Cg*3^iii^	0.97	2.86	3.5375 (2)	128

Symmetry codes: (i) 
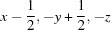
; (ii) 
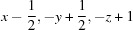
; (iii) 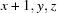.

**Table 2:** Comparison of selected geometric data for (**I**) (Å, °) from X-ray and calculated (DFT) data

	X-ray	B3LYP/6–311G(d,p)
N1—C15	1.272 (3)	1.271
N1—C16	1.468 (4)	1.466
C16—C17	1.528 (4)	1.531
C17—C18	1.499 (4)	1.494
C15—C14	1.479 (4)	1.494
C25—N2	1.372 (3)	1.369
N2—C24	1.371 (4)	1.371
C16—N1—C15	115.2 (2)	115.31
N1—C16—C17	110.4 (2)	110.50
N1—C15—C14	126.3 (3)	126.16
C16—C17—C18	112.2 (2)	112.27

**Table 3:** Experimental details

Crystal data	
Chemical formula	C_25_H_20_N_2_
*M*_r_	348.43
Crystal system, space group	Orthorhombic, *P*2_1_2_1_2_1_
Temperature (K)	100
*a*, *b*, *c* (Å)	6.0044 (3), 16.4721 (7), 17.8957 (9)
*V* (Å^3^)	1769.98 (15)
*Z*	4
Radiation type	Mo *K*α
μ (mm^−1^)	0.08
Crystal size (mm)	0.20 × 0.15 × 0.13

Data collection	
Diffractometer	Bruker SMART APEX CCD
Absorption correction	Multi-scan (*SADABS*; Bruker, 2003 ▸)
*T*_min_, *T*_max_	0.875, 0.990
No. of measured, independent and observed [*I* > 2σ(*I*)] reflections	14169, 3127, 2577
*R*_int_	0.064
(sin θ/λ)_max_ (Å^−1^)	0.596

Refinement	
*R*[*F* ^2^ > 2σ(*F* ^2^)], *wR*(*F* ^2^), *S*	0.041, 0.093, 1.04
No. of reflections	3127
No. of parameters	245
H-atom treatment	H-atom parameters constrained
Δρ_max_, Δρ_min_ (e Å^−3^)	0.15, −0.20
Absolute structure	Refined as an inversion twin

Computer programs: *SMART* and *SAINT* (Bruker, 2003 ▸), *SHELXT* (Sheldrick, 2015*a* ▸), *SHELXTL* (Sheldrick, 2008 ▸), *Mercury* (Macrae *et al.*, 2008 ▸), *SHELXL2016* (Sheldrick, 2015*b* ▸) and *PLATON* (Spek, 2009 ▸).

## supplementary crystallographic information

### Crystal data

**Table:**

C_25_H_20_N_2_	*D*_x_ = 1.308 Mg m^−^^3^
*M**_r_* = 348.43	Mo *K*α radiation, λ = 0.71073 Å
Orthorhombic, *P*2_1_2_1_2_1_	Cell parameters from 5148 reflections
*a* = 6.0044 (3) Å	θ = 2.6–27.4°
*b* = 16.4721 (7) Å	µ = 0.08 mm^−^^1^
*c* = 17.8957 (9) Å	*T* = 100 K
*V* = 1769.98 (15) Å^3^	Needle, yellow
*Z* = 4	0.20 × 0.15 × 0.13 mm
*F*(000) = 736	

### Data collection

**Table:**

Bruker SMART APEX CCD diffractometer	3127 independent reflections
Radiation source: fine-focus sealed tube	2577 reflections with *I* > 2σ(*I*)
Graphite monochromator	*R*_int_ = 0.064
ω scans	θ_max_ = 25.1°, θ_min_ = 2.3°
Absorption correction: multi-scan (SADABS; Bruker, 2003)	*h* = −7→7
*T*_min_ = 0.875, *T*_max_ = 0.990	*k* = −19→19
14169 measured reflections	*l* = −21→21

### Refinement

**Table:**

Refinement on *F*^2^	Secondary atom site location: difference Fourier map
Least-squares matrix: full	Hydrogen site location: inferred from neighbouring sites
*R*[*F*^2^ > 2σ(*F*^2^)] = 0.041	H-atom parameters constrained
*wR*(*F*^2^) = 0.093	*w* = 1/[σ^2^(*F*_o_^2^) + (0.0478*P*)^2^] where *P* = (*F*_o_^2^ + 2*F*_c_^2^)/3
*S* = 1.04	(Δ/σ)_max_ < 0.001
3127 reflections	Δρ_max_ = 0.15 e Å^−^^3^
245 parameters	Δρ_min_ = −0.20 e Å^−^^3^
0 restraints	Absolute structure: Refined as an inversion twin
Primary atom site location: structure-invariant direct methods	

### Special details

**Table:**

Geometry. All esds (except the esd in the dihedral angle between two l.s. planes) are estimated using the full covariance matrix. The cell esds are taken into account individually in the estimation of esds in distances, angles and torsion angles; correlations between esds in cell parameters are only used when they are defined by crystal symmetry. An approximate (isotropic) treatment of cell esds is used for estimating esds involving l.s. planes.

### Fractional atomic coordinates and isotropic or equivalent isotropic displacement parameters (Å^2^)

**Table:**

	*x*	*y*	*z*	*U*_iso_*/*U*_eq_	
N1	0.9441 (4)	0.37168 (13)	0.28719 (12)	0.0239 (6)	
N2	0.6749 (4)	0.30313 (14)	0.06673 (12)	0.0282 (6)	
H2A	0.546902	0.303463	0.045136	0.034*	
C18	0.9807 (5)	0.34812 (16)	0.12665 (15)	0.0239 (7)	
C1	0.5718 (5)	0.20309 (15)	0.32067 (14)	0.0199 (6)	
C8	0.3608 (5)	0.30172 (16)	0.42939 (14)	0.0216 (6)	
C15	0.8796 (5)	0.30043 (16)	0.30408 (14)	0.0219 (6)	
H15	0.975892	0.257990	0.292647	0.026*	
C13	0.5640 (5)	0.32872 (15)	0.39461 (15)	0.0216 (6)	
C14	0.6662 (5)	0.27861 (15)	0.34017 (14)	0.0206 (6)	
C7	0.2732 (5)	0.22659 (16)	0.41038 (14)	0.0235 (7)	
H7	0.144366	0.208937	0.434185	0.028*	
C9	0.2567 (5)	0.35163 (17)	0.48380 (15)	0.0265 (7)	
H9	0.124712	0.334465	0.505954	0.032*	
C19	1.0013 (5)	0.26179 (15)	0.11461 (15)	0.0227 (7)	
C6	0.3713 (5)	0.17673 (16)	0.35700 (14)	0.0226 (7)	
C2	0.6601 (5)	0.15155 (15)	0.26395 (14)	0.0247 (7)	
H2	0.788158	0.167424	0.238604	0.030*	
C5	0.2749 (5)	0.10058 (16)	0.33667 (15)	0.0274 (7)	
H5	0.146320	0.083137	0.360777	0.033*	
C20	1.1624 (5)	0.20388 (17)	0.13290 (15)	0.0256 (7)	
H20	1.293314	0.219244	0.156804	0.031*	
C24	0.8068 (5)	0.23616 (17)	0.07725 (15)	0.0250 (7)	
C23	0.7688 (5)	0.15516 (17)	0.05852 (15)	0.0267 (7)	
H23	0.639991	0.139260	0.033642	0.032*	
C3	0.5619 (5)	0.07970 (16)	0.24594 (16)	0.0276 (7)	
H3	0.624024	0.047503	0.208676	0.033*	
C4	0.3669 (5)	0.05321 (16)	0.28300 (15)	0.0292 (7)	
H4	0.302371	0.003680	0.270620	0.035*	
C12	0.6514 (5)	0.40513 (15)	0.41843 (15)	0.0260 (7)	
H12	0.783119	0.424184	0.397451	0.031*	
C11	0.5466 (5)	0.45039 (17)	0.47086 (15)	0.0294 (7)	
H11	0.607818	0.499841	0.485263	0.035*	
C25	0.7808 (5)	0.36954 (17)	0.09638 (15)	0.0279 (7)	
H25	0.723985	0.422058	0.095887	0.033*	
C16	1.1623 (5)	0.37629 (16)	0.25049 (15)	0.0257 (7)	
H16A	1.234310	0.323625	0.252515	0.031*	
H16B	1.255871	0.414955	0.276677	0.031*	
C10	0.3465 (5)	0.42396 (17)	0.50402 (16)	0.0293 (7)	
H10	0.276083	0.456023	0.539681	0.035*	
C22	0.9296 (5)	0.09975 (18)	0.07835 (16)	0.0312 (7)	
H22	0.908244	0.045184	0.067111	0.037*	
C17	1.1358 (5)	0.40255 (16)	0.16908 (15)	0.0268 (7)	
H17A	1.079262	0.457682	0.167552	0.032*	
H17B	1.280575	0.402183	0.145020	0.032*	
C21	1.1245 (5)	0.12360 (16)	0.11501 (16)	0.0306 (7)	
H21	1.230368	0.084699	0.127540	0.037*	

### Atomic displacement parameters (Å^2^)

**Table:**

	*U*^11^	*U*^22^	*U*^33^	*U*^12^	*U*^13^	*U*^23^
N1	0.0244 (13)	0.0239 (13)	0.0233 (13)	−0.0025 (11)	−0.0001 (11)	0.0016 (10)
N2	0.0235 (13)	0.0334 (14)	0.0277 (12)	0.0037 (13)	−0.0039 (11)	0.0010 (11)
C18	0.0264 (17)	0.0259 (16)	0.0194 (14)	0.0018 (14)	0.0041 (13)	0.0033 (12)
C1	0.0216 (15)	0.0179 (14)	0.0201 (14)	0.0003 (13)	−0.0036 (12)	0.0038 (12)
C8	0.0233 (16)	0.0231 (15)	0.0184 (13)	0.0026 (15)	−0.0044 (12)	0.0048 (12)
C15	0.0233 (16)	0.0198 (15)	0.0225 (14)	0.0034 (14)	−0.0035 (12)	−0.0026 (12)
C13	0.0240 (15)	0.0208 (15)	0.0201 (15)	0.0008 (13)	−0.0051 (12)	0.0042 (11)
C14	0.0213 (15)	0.0215 (15)	0.0190 (13)	0.0002 (13)	−0.0038 (12)	0.0053 (11)
C7	0.0215 (15)	0.0280 (17)	0.0210 (15)	0.0002 (13)	−0.0001 (12)	0.0068 (12)
C9	0.0256 (16)	0.0310 (18)	0.0230 (16)	0.0052 (15)	0.0006 (13)	0.0040 (13)
C19	0.0260 (17)	0.0246 (16)	0.0177 (14)	−0.0010 (14)	0.0037 (13)	−0.0001 (12)
C6	0.0238 (16)	0.0259 (16)	0.0179 (15)	−0.0011 (14)	−0.0046 (12)	0.0048 (11)
C2	0.0244 (15)	0.0245 (15)	0.0252 (15)	0.0009 (14)	0.0032 (13)	0.0041 (12)
C5	0.0305 (17)	0.0260 (16)	0.0257 (15)	−0.0071 (15)	−0.0019 (13)	0.0036 (13)
C20	0.0228 (15)	0.0287 (16)	0.0253 (15)	−0.0012 (15)	0.0002 (13)	−0.0008 (13)
C24	0.0254 (16)	0.0301 (16)	0.0193 (14)	0.0014 (14)	0.0026 (13)	0.0027 (12)
C23	0.0252 (17)	0.0345 (18)	0.0202 (14)	−0.0052 (15)	−0.0006 (12)	−0.0011 (13)
C3	0.0342 (18)	0.0203 (15)	0.0283 (16)	0.0027 (15)	0.0020 (14)	0.0005 (12)
C4	0.0359 (18)	0.0233 (16)	0.0284 (16)	−0.0070 (15)	−0.0023 (15)	0.0015 (13)
C12	0.0274 (16)	0.0237 (15)	0.0270 (15)	−0.0007 (15)	−0.0010 (14)	0.0035 (12)
C11	0.0409 (19)	0.0211 (15)	0.0262 (16)	−0.0018 (15)	−0.0046 (14)	−0.0021 (13)
C25	0.0339 (18)	0.0250 (16)	0.0247 (15)	0.0030 (15)	0.0034 (14)	0.0029 (12)
C16	0.0235 (15)	0.0214 (14)	0.0323 (16)	−0.0059 (14)	−0.0021 (14)	−0.0005 (12)
C10	0.0371 (18)	0.0284 (17)	0.0223 (14)	0.0081 (16)	0.0001 (15)	−0.0008 (12)
C22	0.0411 (19)	0.0263 (16)	0.0264 (16)	−0.0058 (16)	0.0035 (15)	−0.0029 (13)
C17	0.0267 (16)	0.0208 (15)	0.0328 (16)	−0.0011 (14)	0.0063 (14)	0.0026 (13)
C21	0.0352 (18)	0.0270 (17)	0.0296 (16)	0.0055 (15)	0.0037 (15)	0.0014 (13)

### Geometric parameters (Å, º)

**Table:**

N1—C15	1.272 (3)	C2—H2	0.9300
N1—C16	1.468 (4)	C5—C4	1.355 (4)
N2—C24	1.371 (4)	C5—H5	0.9300
N2—C25	1.372 (3)	C20—C21	1.380 (4)
N2—H2A	0.8600	C20—H20	0.9300
C18—C25	1.363 (4)	C24—C23	1.395 (4)
C18—C19	1.444 (3)	C23—C22	1.375 (4)
C18—C17	1.499 (4)	C23—H23	0.9300
C1—C14	1.411 (4)	C3—C4	1.415 (4)
C1—C2	1.426 (3)	C3—H3	0.9300
C1—C6	1.436 (4)	C4—H4	0.9300
C8—C7	1.387 (4)	C12—C11	1.354 (4)
C8—C9	1.420 (4)	C12—H12	0.9300
C8—C13	1.440 (4)	C11—C10	1.409 (4)
C15—C14	1.479 (4)	C11—H11	0.9300
C15—H15	0.9300	C25—H25	0.9300
C13—C14	1.417 (4)	C16—C17	1.528 (4)
C13—C12	1.429 (4)	C16—H16A	0.9700
C7—C6	1.391 (4)	C16—H16B	0.9700
C7—H7	0.9300	C10—H10	0.9300
C9—C10	1.357 (4)	C22—C21	1.398 (4)
C9—H9	0.9300	C22—H22	0.9300
C19—C20	1.397 (4)	C17—H17A	0.9700
C19—C24	1.411 (4)	C17—H17B	0.9700
C6—C5	1.428 (4)	C21—H21	0.9300
C2—C3	1.361 (4)		
			
C15—N1—C16	115.2 (2)	N2—C24—C23	130.0 (3)
C24—N2—C25	108.7 (2)	N2—C24—C19	107.6 (2)
C24—N2—H2A	125.7	C23—C24—C19	122.4 (3)
C25—N2—H2A	125.7	C22—C23—C24	117.3 (3)
C25—C18—C19	105.7 (3)	C22—C23—H23	121.4
C25—C18—C17	126.4 (2)	C24—C23—H23	121.4
C19—C18—C17	127.7 (3)	C2—C3—C4	121.1 (3)
C14—C1—C2	123.5 (2)	C2—C3—H3	119.5
C14—C1—C6	119.4 (2)	C4—C3—H3	119.5
C2—C1—C6	117.0 (2)	C5—C4—C3	119.5 (3)
C7—C8—C9	121.2 (3)	C5—C4—H4	120.3
C7—C8—C13	119.4 (2)	C3—C4—H4	120.3
C9—C8—C13	119.4 (2)	C11—C12—C13	121.4 (3)
N1—C15—C14	126.3 (3)	C11—C12—H12	119.3
N1—C15—H15	116.9	C13—C12—H12	119.3
C14—C15—H15	116.9	C12—C11—C10	121.2 (3)
C14—C13—C12	124.0 (3)	C12—C11—H11	119.4
C14—C13—C8	119.0 (2)	C10—C11—H11	119.4
C12—C13—C8	117.0 (2)	C18—C25—N2	110.8 (2)
C1—C14—C13	120.7 (2)	C18—C25—H25	124.6
C1—C14—C15	117.0 (2)	N2—C25—H25	124.6
C13—C14—C15	122.3 (2)	N1—C16—C17	110.4 (2)
C8—C7—C6	122.3 (3)	N1—C16—H16A	109.6
C8—C7—H7	118.8	C17—C16—H16A	109.6
C6—C7—H7	118.8	N1—C16—H16B	109.6
C10—C9—C8	121.1 (3)	C17—C16—H16B	109.6
C10—C9—H9	119.5	H16A—C16—H16B	108.1
C8—C9—H9	119.5	C9—C10—C11	119.9 (3)
C20—C19—C24	118.7 (2)	C9—C10—H10	120.1
C20—C19—C18	134.2 (3)	C11—C10—H10	120.1
C24—C19—C18	107.1 (2)	C23—C22—C21	121.5 (3)
C7—C6—C5	121.5 (2)	C23—C22—H22	119.3
C7—C6—C1	119.2 (2)	C21—C22—H22	119.3
C5—C6—C1	119.3 (2)	C18—C17—C16	112.2 (2)
C3—C2—C1	121.7 (3)	C18—C17—H17A	109.2
C3—C2—H2	119.2	C16—C17—H17A	109.2
C1—C2—H2	119.2	C18—C17—H17B	109.2
C4—C5—C6	121.4 (3)	C16—C17—H17B	109.2
C4—C5—H5	119.3	H17A—C17—H17B	107.9
C6—C5—H5	119.3	C20—C21—C22	121.1 (3)
C21—C20—C19	119.1 (3)	C20—C21—H21	119.5
C21—C20—H20	120.5	C22—C21—H21	119.5
C19—C20—H20	120.5		
			
C16—N1—C15—C14	−179.0 (2)	C7—C6—C5—C4	177.9 (3)
C7—C8—C13—C14	1.7 (3)	C1—C6—C5—C4	−1.0 (4)
C9—C8—C13—C14	−179.8 (2)	C24—C19—C20—C21	−1.1 (4)
C7—C8—C13—C12	−177.6 (2)	C18—C19—C20—C21	177.4 (3)
C9—C8—C13—C12	0.9 (3)	C25—N2—C24—C23	178.3 (3)
C2—C1—C14—C13	176.9 (2)	C25—N2—C24—C19	−0.1 (3)
C6—C1—C14—C13	−0.5 (4)	C20—C19—C24—N2	179.1 (2)
C2—C1—C14—C15	−5.9 (4)	C18—C19—C24—N2	0.2 (3)
C6—C1—C14—C15	176.6 (2)	C20—C19—C24—C23	0.6 (4)
C12—C13—C14—C1	178.7 (2)	C18—C19—C24—C23	−178.3 (3)
C8—C13—C14—C1	−0.5 (4)	N2—C24—C23—C22	−177.8 (3)
C12—C13—C14—C15	1.8 (4)	C19—C24—C23—C22	0.3 (4)
C8—C13—C14—C15	−177.5 (2)	C1—C2—C3—C4	0.1 (4)
N1—C15—C14—C1	147.8 (3)	C6—C5—C4—C3	−0.3 (4)
N1—C15—C14—C13	−35.1 (4)	C2—C3—C4—C5	0.8 (4)
C9—C8—C7—C6	179.6 (2)	C14—C13—C12—C11	−179.9 (3)
C13—C8—C7—C6	−1.9 (4)	C8—C13—C12—C11	−0.6 (4)
C7—C8—C9—C10	177.9 (2)	C13—C12—C11—C10	−0.1 (4)
C13—C8—C9—C10	−0.6 (4)	C19—C18—C25—N2	0.3 (3)
C25—C18—C19—C20	−178.9 (3)	C17—C18—C25—N2	−175.1 (2)
C17—C18—C19—C20	−3.6 (5)	C24—N2—C25—C18	−0.2 (3)
C25—C18—C19—C24	−0.3 (3)	C15—N1—C16—C17	111.3 (3)
C17—C18—C19—C24	175.0 (3)	C8—C9—C10—C11	−0.1 (4)
C8—C7—C6—C5	−178.2 (2)	C12—C11—C10—C9	0.5 (4)
C8—C7—C6—C1	0.8 (4)	C24—C23—C22—C21	−0.7 (4)
C14—C1—C6—C7	0.5 (4)	C25—C18—C17—C16	115.4 (3)
C2—C1—C6—C7	−177.2 (2)	C19—C18—C17—C16	−59.1 (4)
C14—C1—C6—C5	179.4 (2)	N1—C16—C17—C18	−55.6 (3)
C2—C1—C6—C5	1.8 (4)	C19—C20—C21—C22	0.7 (4)
C14—C1—C2—C3	−178.9 (3)	C23—C22—C21—C20	0.2 (4)
C6—C1—C2—C3	−1.3 (4)		

### Hydrogen-bond geometry (Å, º)

*Cg*3, *Cg*4 and *Cg*5 are the centroids rings C1/C6–C8/C13/C14, C8–C13 and C19–C24, respectively.

**Table:**

*D*—H···*A*	*D*—H	H···*A*	*D*···*A*	*D*—H···*A*
C12—H12···N1	0.93	2.36	2.9845 (2)	124
C2—H2···*Cg*5	0.93	2.77	3.5505 (2)	142
N2—H2*A*···*Cg*5^i^	0.86	2.59	3.1855 (2)	127
C7—H7···*Cg*4^ii^	0.93	2.75	3.5777 (2)	148
C9—H9···*Cg*3^ii^	0.93	2.73	3.5077 (2)	142
C16—H16*A*···*Cg*3^iii^	0.97	2.86	3.5375 (2)	128

Symmetry codes: (i) *x*−1/2, −*y*+1/2, −*z*; (ii) *x*−1/2, −*y*+1/2, −*z*+1; (iii) *x*+1, *y*, *z*.
